# A Rare Phenotype of Uncommon Charcot–Marie–Tooth Genotypes Complicated With Inflammation Evaluated by Genetics and Magnetic Resonance Neurography

**DOI:** 10.3389/fgene.2022.873641

**Published:** 2022-07-07

**Authors:** Xiaoyun Su, Xiangquan Kong, Zuneng Lu, Lixia Wang, Chuansheng Zheng

**Affiliations:** ^1^ Department of Radiology, Union Hospital, Tongji Medical College, Huazhong University of Science and Technology, Wuhan, China; ^2^ Hubei Province Key Laboratory of Molecular Imaging, Wuhan, China; ^3^ Department of Neurology, Renming Hospital of Wuhan University, Wuhan, China

**Keywords:** Charcot–Marie–Tooth, MPZ gene, next-generation sequencing, magnetic resonance neurography, neuroinflammation

## Abstract

The pathogenesis of Charcot–Marie–Tooth (CMT) disease, an inherited peripheral neuropathy, is associated with more than 60 nuclear genes. We reported a rare phenotype of the uncommon CMT genotype complicated with neuroinflammation, that is, an MPZ mutation, NC_000001.11 (NM_000530.6): c.308G > C detected by next-generation sequencing. Moreover, we present a case of the CMT type 1B, with atypical presentation as two patterns of hypertrophy in the brachial and lumbosacral plexus, as well as enhancement in the cauda equina and nerve roots on multimodal magnetic resonance neurography (MRN). MRN assessment facilitated the identification of coexisting neuroinflammation and provided more evidence, especially for patients with atypical symptoms in hereditary sensory and motor neuropathy, who could benefit from immunotherapy.

## Introduction

Charcot–Marie–Tooth disease (CMT) is a peripheral nerve, single gene, inherited condition with defined clinical and genetic heterogeneity (El-Abassi et al., 2014). It has different incidence rates across various regions and subtypes (Barreto et al., 2016) and its diagnosis is based on a combination of family genetic history, electromyography (EMG), and clinical factors, such as chronic progressive lower extremity distal weakness and muscular atrophy, a reduction in reflexes, and multiple malformations (Pareyson et al., 2006; Barisic et al., 2008; Banchs et al., 2009). Magnetic resonance neuropathy (MRN) has allowed for the accurate, large-scale, three-dimensional display of the entire nerve tract and its branches, furthermore, providing noninvasive quantitative characteristics.

In this study, we reported a rare CMT genotype, a rare MPZ missense mutation, NC_000001.11 (NM_000530.6): c.308G > C that is detected by next-generation sequencing. Moreover, we described a typical presentation as two patterns of hypertrophy in the brachial and lumbosacral (LS) plexus, as well as enhancement in cauda equina and nerve roots, which could be helpful for identifying coexisting inflammation in hereditary sensory and motor neuropathy.

## Materials and Methods

This study was approved by the institutional ethical review board, and a written informed consent was obtained from the patient. Clinical data were obtained from electronic medical records. The multimodal MRN examination was performed on a 3.0T MRI scanner (MAGNETOM Trio, Siemens Healthcare, Germany). The MRN protocol and post-processing procedures were as previously described (Su et al., 2019; Su et al., 2021). Specific sequences parameters are provided in the Supplementary Table.

### Targeted Next-Generation Sequencing

A five ml of venous blood sample was collected from the patient, ultrasonically interrupted, and a DNA library was prepared. The DNA encoding the target gene coding region and the adjacent shear region was enriched using a capture chip, and the mutation was detected by a high-throughput sequencing platform. The proband was tested for the 115 diseased-related genes, such as those associated with CMT, hereditary sensory, and autonomic spastic paraplegia, among other conditions, including the common PMP22, MPZ, and GJBl genes involved in CMT (Hubei Huada Gene Research Institute). Specific sequencing methods are provided in the Supplementary File.

## Results

### Case

A 20-year-old female presented to our neurology outpatient department with easy bruising and fatigue since childhood, which aggravated the weakness of extremities, numbness below the knee joints, and imbalances with recurrent falls in 3 months. The neurological examination showed the disappearance of tendinous reflexes, positive Romberg’s sign, and the muscle force in lower extremities was of grade 4. There was evidence of a bow-shaped foot. However, there were no specific neurological symptoms or signs that were found in the proband’s parents as well as other family members. Furthermore, no evidence of neuropathy was revealed by MRN or EMG examinations in the proband’s parents. Therefore, there was no clear family history in this case. Laboratory evaluation revealed that blood routine, biochemical indices, rheumatoid factors, thyroid, and renal functions were all within normal ranges. Nerve conduction velocity was significantly reduced in all extremities, especially in the right lower extremity. EMG showed denervated patterns and F-wave was not elicited. Lumbar MRI revealed the hypertrophy of the cauda equina.

### Next-Generation Sequencing

**FIGURE 1:**
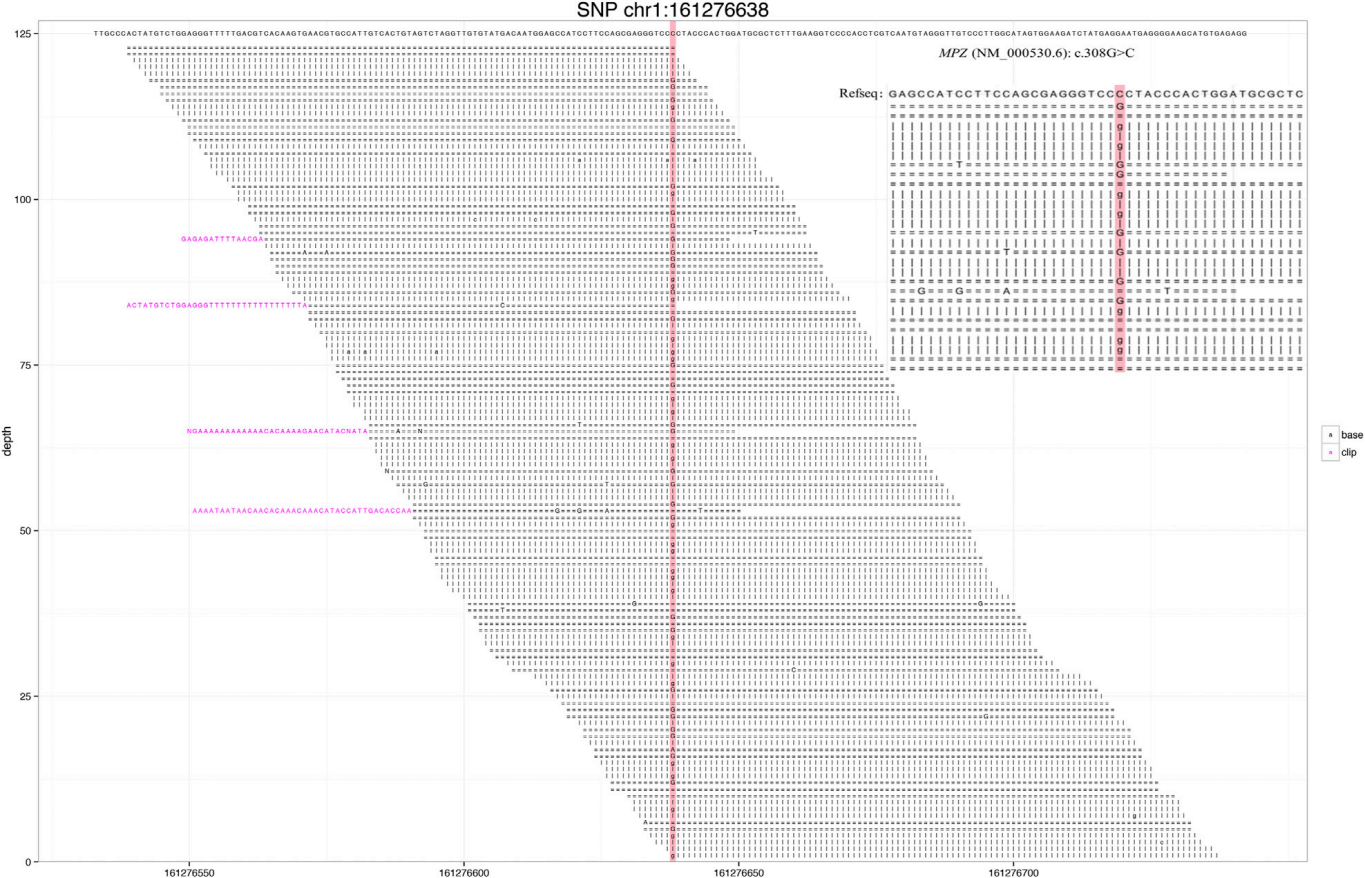
**N**ext-generation sequencing revealed a small number of reads in the myelin protein zero (MPZ) gene. Red area indicates the locus with substitution mutation (c.308G > C), as supported by 171 high-quality reads.

Through gene sequencing, a rare MPZ missense mutation, NC_000001.11 (NM_000530.6): c.308G > C was detected **(** Figure 1), and the case was diagnosed as the CMT type 1B. Based on previous reports, Sanger sequencing validation was not performed for our case because the number of reads at site was >40X and the ratio >40% (Strom et al., 2014; Arteche-Lopez et al., 2021).

### MRN Characteristics

**FIGURE 2:**
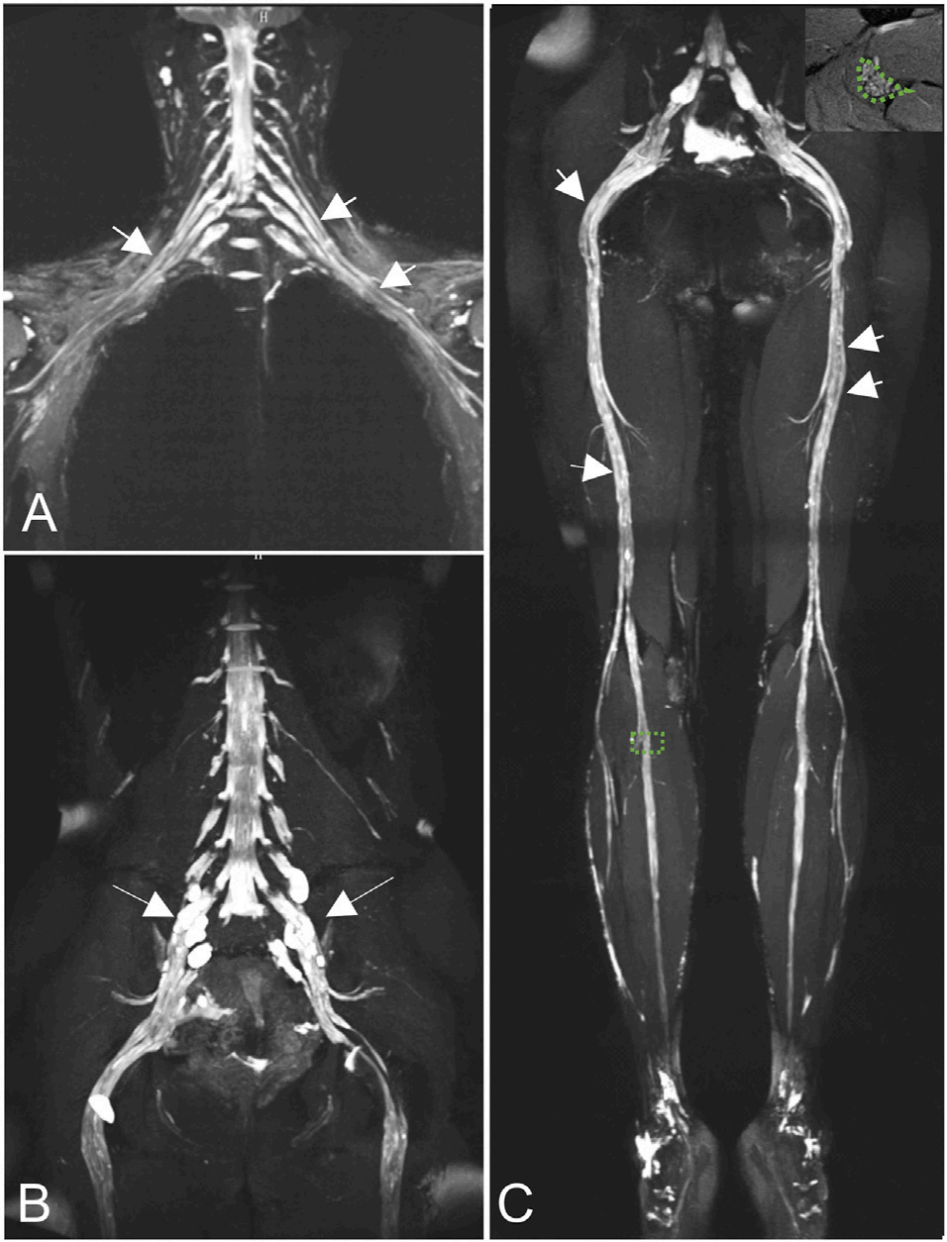
Coronal magnetic resonance neurography (MRN) showed that diffuse uniform hypertrophy of the brachial plexus **(A)** and multifocal fusiform hypertrophy of the lumbosacral (LS) plexus [**(B)**, long arrows)], with increased signal intensity. Neural stems exhibited *worm-eaten* signal reductions in the brachial, LS plexus, and sciatic nerves (short arrows). Magnified views of areas of interest on the right peroneal nerve are shown in the right upper corners of the images **(C).**

**FIGURE 3:**
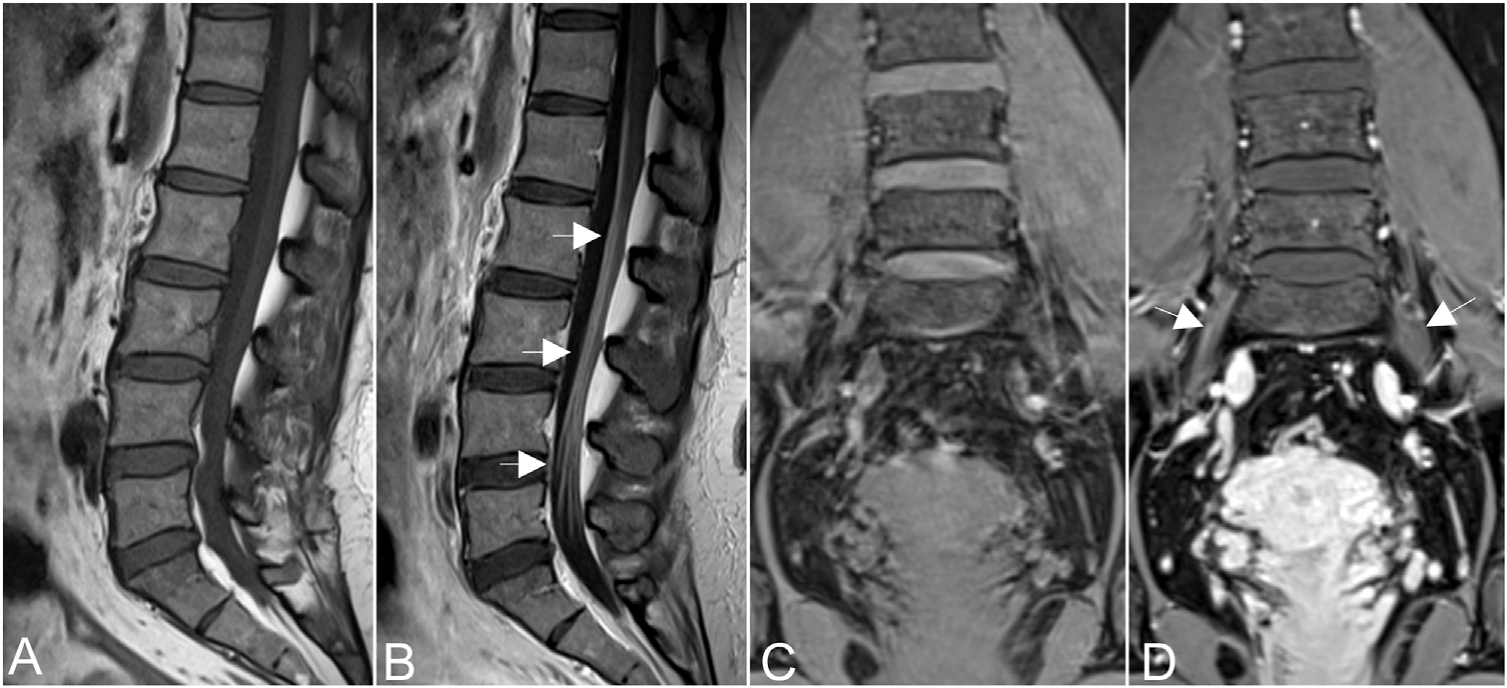
Obvious enhancement in the cauda equina [arrows in **(A,B)**], and mild enhancements were observed in the nerve roots of the hypertrophic plexus (arrows in **(C,D)**] on T1-weighted images after gadolinium administration.

**FIGURE 4:**
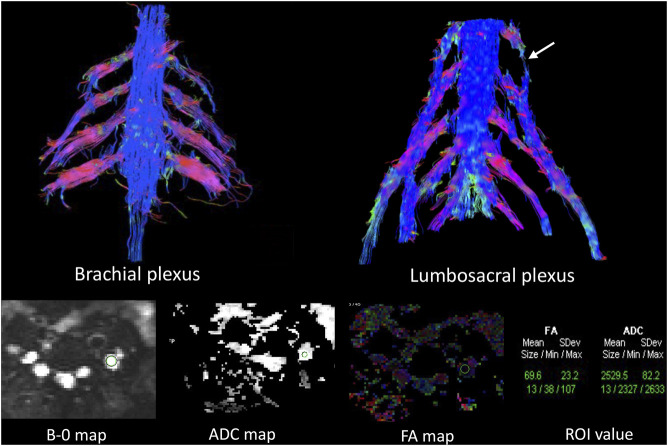
Diffusion tensor tractography (DTT) revealed significantly thickened and distorted discontinuous tracts in the brachial and lumbosacral (LS) plexus. The FA value was significantly decreased, while the ADC value was increased in the plexus. The left L4 nerve roots were almost interrupted in DTT (arrows in the LS plexus), corresponding to fusiform hypertrophy, the site in which the FA (0.069) and ADC values (2.529) were close to the diffusion weighting of the free water.

MRN showed that nerve trunks of the entire body had prominent hypertrophy, including brachial and LS plexus as well as its branches **(** Figure 2). The nerve roots of the LS plexus exhibited multifocal fusiform hypertrophy with hyperintensity on T2-weighted image (T2WI), suggesting a high-water content. It should be noted that the neural stems exhibited inhomogeneous and focal hypointense, as *worm-eaten* cavities, were observed in the LS plexus and sciatic nerves **(** Figure 2). Furthermore, after gadolinium administration, obvious enhancement in the cauda equina and mild enhancements were observed in the nerve roots of the LS plexus on T1-weighted images **(** Figure 3). Diffusion tensor imaging (DTI) revealed that the fractional anisotropy (FA) value was significantly decreased by about 0.1 (normal range, 0.4–0.7), while the corresponding apparent diffusion coefficient (ADC) value increased. The left L4 nerve root was nearly interrupted, corresponding to the site of fusiform hypertrophy **(** Figure 4). Brain perfusion SPECT revealed slightly decreased bilateral cerebellar blood perfusion (Supplementary Figure).

A lumbar puncture examination revealed that the cerebrospinal fluid was clear with total protein concentrations of 0.74 g/L. Coexisting neuroinflammation was suspected by the neurologist, upon which experimental treatment with intravenous immunoglobulin was administered. After 1 month, neuromuscular symptoms gradually improved to a certain extent. Finally, the patient could walk unaided with recovered muscle force at 5 grades in lower extremities, accompanied by mild ataxia.

## Discussion

In this study, we report a case of a rare MPZ mutation with an uncommon phenotype, that NC_000001.11 (NM_000530.6): c.308G > C was detected by next-generation sequencing. The SIFT and PolyPhen-2 databases were used to predict the variability in the heterozygote, and the incidence was very low in the population. In 2001, Fabrizi et al. (2001)first found the c.308G > A mutation in two CMT type I patients who were sisters, and verified that the mutation was not present in 50 unaffected individuals. The MPZ is located at locus 1q22 and belongs to the immunoglobulin superfamily and it is a type of adhesion molecule connecting sphingomyelin and plays a role in the formation of the myelin sheath. Most mutations lead to the formation of an abnormal extracellular structure of the protein (Warner et al., 1996; Shy et al., 2004).

MRN revealed hypertrophy of the nerve roots and their branches in the entire body, accompanied by increased T2 signal intensity, which was due to repeated demyelination and remyelination, consistent with previous findings (Chung et al., 2008; Chhabra et al., 2016; Khadilkar et al., 2017). Chhabra et al. (2016) reported that nerves in CMT show predominantly diffuse thickening, whereas those in chronic inflammatory demyelinating polyneuropathy (CIDP) are discontinuous, non-uniform, or patchily thickened. We found diffuse uniform hypertrophy of the brachial plexus, and multifocal fusiform hypertrophy of the LS plexus. The thickened pattern was similar to that of CIDP (Su et al., 2020). The inherent mechanism should be evaluated further.

As early as 1982, there were reports of the coexistence of hereditary and inflammatory neuropathy (Dyck et al., 1982). Gabriel et al. (2002) reported neuroinflammation in some CMT type I patients, confirmed by nerve biopsy , which is an invasive examination. In this case, EMG revealed severe diffuse sensorimotor polyneuropathy with demyelination and axonal loss, but failed to identify inflammation. When CMT is combined with certain types of acute and chronic peripheral nerve inflammation, clinical and electrophysiological examinations can be even more difficult to resolve, with potentially missed diagnoses (Trivedi et al., 2015; Zhan et al., 2015). However, enhancement was found in the cauda equina and nerve roots, implying coexistent inflammation, resulting from the increased permeability of the BNB. Moreover, the signal reductions, that is *worm-eaten cavit*ies, were detected in neural stems, which may be attributed to the effects of short T2 relaxation time by contrast agents, a feature that has been reported in CIDP patients (Su et al., 2020). Subsequent neuroinflammation, in this case, was confirmed by experimental immunotherapy and lumbar puncture.

DTI, a relatively powerful MR technology for nerve tracer imaging, provides quantitative assessment of peripheral nerve degeneration and changes in the tissue microstructure (Mukherjee et al., 2008; Tagliafico et al., 2011). It is generally believed that FA values are correlated with the density and diameter of axons as well as the density and thickness of myelin sheath, though the relation is thought to be much closer to the axons. When genetics lead to abnormal development of the myelin, long-term repeated loss and repair of the myelin sheath and axonal degeneration cause the cessation of diffusion of water molecules along the long axis of the nerve, and the FA value decreases (Carvalho et al., 2005). In this case, the site of fusiform hypertrophy in the LS plexus has close to the diffusion weighting of free water, indicating severe destruction of nerve tracts of the myelin sheath. This outcome is consistent with that of previous EMG studies; however, it is not consistent with mild clinical neurological signs after immunotherapy, which may probably be related to compensation due to slow progression of nerve injury over a long time period.

This study has some limitations. First, we failed to acquire the MRN characteristics of CMT type 1B with MPZ mutation while without neuroinflammation as a comparison. We have no more patients with CMT type IB disease associated with MPZ mutations, due to the incidence being very low in the population. Second, since there was only one case, we could not make a correlation analysis between the MRN pattern and disease duration. Therefore, a larger sample size might be required in future studies.

In conclusion, we reported a rare CMT genotype with atypical presentation complicated with neuroinflammation. MRN may be vital for identifying coexisting inflammation in hereditary sensory and motor neuropathy, especially for the patients with atypical symptoms or lack of a clear history. Furthermore, genotyping can provide scientific guidance for early intervention while prenatal DNA testing or pre-implantation genome diagnosis is important to protect future generations.

## Data Availability

The datasets for this article are not publicly available due to concerns regarding participant/patient anonymity. Requests to access the datasets should be directed to the corresponding authors.

## References

[B1] Arteche-López A. Ávila-Fernández A. Romero R. Riveiro-Álvarez R. López-Martínez M. A. Giménez-Pardo A. (2021). Sanger Sequencing Is No Longer Always Necessary Based on a Single-Center Validation of 1109 NGS Variants in 825 Clinical Exomes. Sci. Rep. 11, 5697. 10.1038/s41598-021-85182-w 33707547 PMC7952542

[B2] Banchs I. Casasnovas C. Albertí A. De Jorge L. Povedano M. Montero J. (2009). Diagnosis of Charcot-Marie-Tooth Disease. J. Biomed. Biotechnol. 2009, 985415. 10.1155/2009/985415 19826499 PMC2760395

[B3] Barisic N. Claeys K. G. Sirotković-Skerlev M. Löfgren A. Nelis E. De Jonghe P. (2008). Charcot-Marie-Tooth Disease: a Clinico-Genetic Confrontation. Ann. Hum. Genet. 72, 416–441. 10.1111/j.1469-1809.2007.00412.x 18215208

[B4] Barreto L. C. L. S. Oliveira F. S. Nunes P. S. de França Costa I. M. P. Garcez C. A. Goes G. M. (2016). Epidemiologic Study of Charcot-Marie-Tooth Disease: A Systematic Review. Neuroepidemiology 46, 157–165. 10.1159/000443706 26849231

[B5] Carvalho A. A. S. Vital A. Ferrer X. Latour P. Lagueny A. Brechenmacher C. (2005). Charcot-Marie-Tooth Disease Type 1A: Clinicopathological Correlations in 24 Patients. J. Peripher Nerv. Syst. 10, 85–92. 10.1111/j.1085-9489.2005.10112.x 15703022

[B6] Chhabra A. Carrino J. A. Farahani S. J. Thawait G. K. Sumner C. J. Wadhwa V. (2016). Whole-body MR Neurography: Prospective Feasibility Study in Polyneuropathy and Charcot-Marie-Tooth Disease. J. Magn. Reson. Imaging 44, 1513–1521. 10.1002/jmri.25293 27126998

[B7] Chung K. W. Suh B. C. Shy M. E. Cho S. Y. Yoo J. H. Park S. W. (2008). Different Clinical and Magnetic Resonance Imaging Features between Charcot-Marie-Tooth Disease Type 1A and 2A. Neuromuscul. Disord. 18, 610–618. 10.1016/j.nmd.2008.05.012 18602827

[B8] Dyck P. J. Swanson C. J. Low P. A. Bartleson J. D. Lambert E. H. (1982). Prednisone-responsive Hereditary Motor and Sensory Neuropathy. Mayo Clin. Proc. 57, 239–246. 7070119

[B9] El-Abassi R. England J. D. Carter G. T. (2014). Charcot-Marie-Tooth Disease: an Overview of Genotypes, Phenotypes, and Clinical Management Strategies. PM&R 6, 342–355. 10.1016/j.pmrj.2013.08.611 24434692

[B10] Fabrizi G. M. Ferrarini M. Cavallaro T. Jarre L. Polo A. Rizzuto N. (2001). A Somatic and Germline Mosaic Mutation in MPZ/P0 Mimics Recessive Inheritance of CMT1B. Neurology 57, 101–105. 10.1212/wnl.57.1.101 11445635

[B11] Gabriel C. M. Gregson N. A. Wood N. W. Hughes R. A. (2002). Immunological Study of Hereditary Motor and Sensory Neuropathy Type 1a (HMSN1a). J. Neurol. Neurosurg. Psychiatry 72, 230–235. 10.1136/jnnp.72.2.230 11796774 PMC1737757

[B12] Khadilkar S. Patil N. Kadam N. Mansukhani K. Patel B. (2017). Clinico-Electrophysiological and Genetic Overlaps and Magnetic Resonance Imaging Findings in Charcot-Marie- Tooth Disease: A Pilot Study from Western India. Ann. Indian Acad. Neurol. 20, 425–429. 10.4103/aian.aian_316_17 29184351 PMC5682752

[B13] Mukherjee P. Berman J. I. Chung S. W. Hess C. P. Henry R. G. (2008). Diffusion Tensor MR Imaging and Fiber Tractography: Theoretic Underpinnings. AJNR Am. J. Neuroradiol. 29, 632–641. 10.3174/ajnr.a1051 18339720 PMC7978191

[B14] Pareyson D. Scaioli V. Laurà M. (2006). Clinical and Electrophysiological Aspects of Charcot-Marie-Tooth Disease. Neuromol Med. 8, 3–22. 10.1385/nmm:8:1-2:3 16775364

[B15] Shy M. E. Jani A. Krajewski K. Grandis M. Lewis R. A. Li J. (2004). Phenotypic Clustering in MPZ Mutations. Brain 127, 371–384. 10.1093/brain/awh048 14711881

[B16] Strom S. P. Lee H. Das K. Vilain E. Nelson S. F. Grody W. W. (2014). Assessing the Necessity of Confirmatory Testing for Exome-Sequencing Results in a Clinical Molecular Diagnostic Laboratory. Genet. Med. 16, 510–515. 10.1038/gim.2013.183 24406459 PMC4079763

[B17] Su X. Kong X. Alwalid O. Wang J. Zhang H. Lu Z. (2021). Multisequence Quantitative Magnetic Resonance Neurography of Brachial and Lumbosacral Plexus in Chronic Inflammatory Demyelinating Polyneuropathy. Front. Neurosci. 15, 649071. 10.3389/fnins.2021.649071 34366769 PMC8346234

[B18] Su X. Kong X. Liu D. Kong X. Alwalid O. Wang J. (2019). Multimodal Magnetic Resonance Imaging of Peripheral Nerves: Establishment and Validation of Brachial and Lumbosacral Plexi Measurements in 163 Healthy Subjects. Eur. J. Radiology 117, 41–48. 10.1016/j.ejrad.2019.05.017 31307651

[B19] Su X. Kong X. Lu Z. Zhou M. Wang J. Liu X. (2020). Use of Magnetic Resonance Neurography for Evaluating the Distribution and Patterns of Chronic Inflammatory Demyelinating Polyneuropathy. Korean J. Radiol. 21, 483–493. 10.3348/kjr.2019.0739 32193896 PMC7082655

[B20] Tagliafico A. Calabrese M. Puntoni M. Pace D. Baio G. Neumaier C. E. (2011). Brachial Plexus MR Imaging: Accuracy and Reproducibility of DTI-Derived Measurements and Fibre Tractography at 3.0-T. Eur. Radiol. 21, 1764–1771. 10.1007/s00330-011-2100-z 21424901

[B21] Trivedi J. R. Phillips L. Chhabra A. (2015). Hereditary and Acquired Polyneuropathy Conditions of the Peripheral Nerves: Clinical Considerations and MR Neurography Imaging. Semin. Musculoskelet. Radiol. 19, 130–136. 10.1055/s-0035-1545076 25764237

[B22] Warner L. E. Hilz M. J. Appel S. H. Killian J. M. Kolodny E. H. Karpati G. (1996). Clinical Phenotypes of Different MPZ (P0) Mutations May Include Charcot-Marie-Tooth Type 1B, Dejerine-Sottas, and Congenital Hypomyelination. Neuron 17, 451–460. 10.1016/s0896-6273(00)80177-4 8816708

[B23] Zhan Y. Zi X. Hu Z. Peng Y. Wu L. Li X. (2015). PMP22-Related Neuropathies and Other Clinical Manifestations in Chinese Han Patients with Charcot-Marie-Tooth Disease Type 1. Muscle Nerve 52, 69–75. 10.1002/mus.24550 25522693

