# Supplementary material for: A Rare Phenotype of Uncommon Charcot–Marie–Tooth Genotypes Complicated With Inflammation Evaluated by Genetics and Magnetic Resonance Neurography
Source: Front Genet. 2022 Jul 7;13:873641. doi: 10.3389/fgene.2022.873641 (PMC9302481; doi:10.3389/fgene.2022.873641)
Supplement: Supplementary file 1 [file Presentation1.pdf]

Supplementary Table [1]

Sequences	SPACE	PDWI	VIBE	DTI
**TR (ms)**	3000	6000	4000	6000
**TE (ms)**	270	92	39	92
**Avgerage**	1.8	3	2	4
**Slices**	144	45	37	45
**FOV (mm^2)**	448×448^2	420×420^2	448×448^2	256×256^2
**Voxel (mm^3)**	1.0×1.0×1.0	2.0×2.0×3.0	1.0×1.0×1.0	2.0×2.0×3.0
**Bandwidth (Hz/px)**	425	266	250	1345
**IPAT**	3	2	2	2
**Time (min)**	11’14	3’40	2’20	5’32

[1] FOV, field of view; IPAT, integrated parallel acquisition technology; PX, pixel; SPACE: sampling perfection with application-optimized contrasts using different flip angle evolution; PDWI, proton density weighted image; VIBE: volumetric interpolated breath-hold examination; DTI: diffusion tensor imaging.

Materials And Methods

Whole exome sequencing and mutation selection

Genomic DNA was extracted from peripheral blood using a QIAamp DNA Blood Mini Kit (Qiagen, Hilden, Germany) according to the manufacturer's instructions. All three family members (parents, proband) were subjected to exome sequencing. Sequences were captured by Agilent SureSelect version 4 (Agilent Technologies, Santa Clara, CA) according to the manufacturer's protocols. The enriched library was sequenced on an Illumina HighSeq2000. The sequencing reads were aligned to GRCh37.p10 using Burrows-Wheeler Aligner software (version 0.59). We then performed local realignment and base quality recalibration of the Burrows-Wheeler aligned reads using the GATK IndelRealigner and the GATK BaseRecalibrator, respectively (broadinstitute.org/). Single-nucleotide variants (SNV) and small insertions or deletions (indel) were identified by the GATK UnifiedGenotyper (broadinstitute.org/). Variants were annotated using the Consensus Coding Sequences Database (20130630) at the National Center for Biotechnology Information.

Targeted exome-based next-generation sequencing and variant identification

DNA samples obtained from the proband (III-1) were sequenced using target exome-based next-generation sequencing. Roche NimbleGen's (Madison, USA) custom Sequence Capture 2.1M Human Array was used to designed to capture a 139 kb comprising all exons (including the 100 bp of introns) of APC genes which is associated with FAP and CRC. The procedure for preparation of libraries was consistent with standard operating protocols published previously. In each pooling batch, 10 to 33

samples were sequenced simultaneously on IlluminaHiSeq 2500 Analyzers (Illumina, San Diego, USA) for 90 cycles. Image analysis, error estimation, and base calling were performed using Illumina Pipeline software (version 1.3.4) to generate raw data. The raw reads were screened to generate – clean reads‖ followed by established filtering criteria. Clean reads with a length of 90 bp were aligned to the reference human genome from the NCBI database (Build 37) using the Burrows Wheeler Aligner (BWA) Multi-Vision software package with output files in - bam‖ format. The bam data were used for reads coverage in the target region and sequencing depth computation, SNP and INDEL calling, and CNV detection. First, a novel three-step computational framework for CNV was applied (**As shown in the framework below**). Then, SNPs and INDELs were called using SOAPsnp software and Sam tools pileup software, respectively.

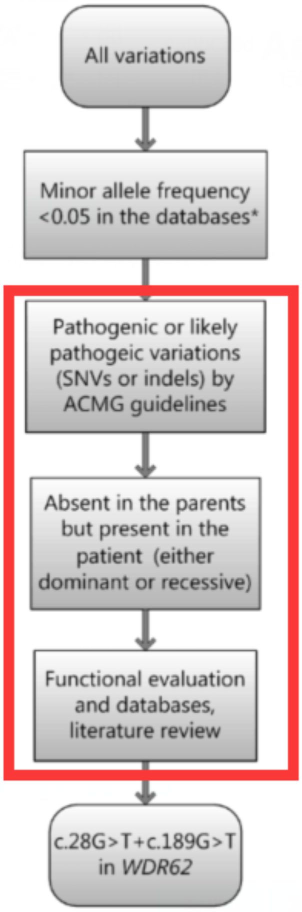

Supplementary Figure

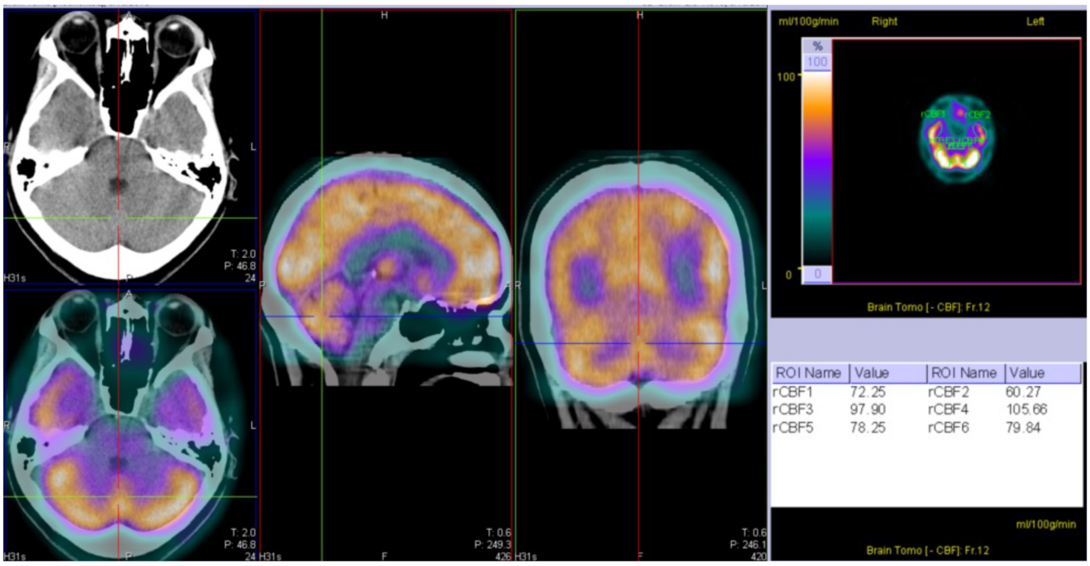

Brain perfusion SPECT revealed that bilateral cerebellar blood perfusion slightly decreased (rCBF, left 79.84ml/100g/min, right 78.25 ml/100g/min).

Unrestricted
